# The ischemic preconditioning effect of adenosine in patients with ischemic heart disease

**DOI:** 10.1186/1476-7120-7-52

**Published:** 2009-11-05

**Authors:** Bita Sadigh, Miguel Quintana, Christer Sylvén, Margareta Berglund, Lars Åke Brodin

**Affiliations:** 1 The Karolinska Institute, at the Department of Cardiology, Karolinska University Hospital, Huddinge, Stockholm, Sweden; 2 Institution of Laboratory Medicine, Department of Cardiology, Hospital de Torrevieja, Alicante, Spain; 3 Institution of Laboratory Medicine, Karolinska University Hospital, Huddinge, Stockholm, Sweden

## Abstract

**Introduction:**

*In vivo *and *in vitro *evidence suggests that adenosine and its agonists play key roles in the process of ischemic preconditioning. The effects of low-dose adenosine infusion on ischemic preconditioning have not been thoroughly studied in humans.

**Aims:**

We hypothesised that a low-dose adenosine infusion could reduce the ischemic burden evoked by physical exercise and improve the regional left ventricular (LV) systolic function.

**Materials and methods:**

We studied nine severely symptomatic male patients with severe coronary artery disease. Myocardial ischemia was induced by exercise on two separate occasions and quantified by Tissue Doppler Echocardiography. Prior to the exercise test, intravenous low-dose adenosine or placebo was infused over ten minutes according to a randomized, double blind, cross-over protocol. The LV walls were defined as ischemic if a reduction, no increment, or an increment of < 15% in peak systolic velocity (PSV) was observed during maximal exercise compared to the baseline values observed prior to placebo-infusion. Otherwise, the LV walls were defined as non-ischemic.

**Results:**

PSV increased from baseline to maximal exercise in non-ischemic walls both during placebo (*P *= 0.0001) and low-dose adenosine infusion (*P *= 0.0009). However, in the ischemic walls, PSV increased only during low-dose adenosine infusion *(P *= 0.001), while no changes in PSV occurred during placebo infusion (*P *= NS).

**Conclusion:**

Low-dose adenosine infusion reduced the ischemic burden and improved LV regional systolic function in the ischemic walls of patients with exercise-induced myocardial ischemia, confirming that adenosine is a potential preconditioning agent in humans.

## Introduction

Ischemic preconditioning protects the heart through brief episodes of ischemia and renders the myocardium resistant to subsequent ischemic insults. The potential beneficial effects of ischemic preconditioning include the preservation of the left ventricular (LV) systolic and diastolic functions[1], the preservation of adenosine triphosphate levels in myocardial biopsy specimens[1], and the prevention of arrhythmias by minimizing QT and JT dispersion[2].

Ischemic preconditioning is triggered by the binding of several humoral factors such as adenosine, bradykinin, and opioids to their cardiac receptors[3,4], thereby initiating a series of complex and divergent signal cascades that eventually converge on protein kinase C. The cardioprotective effect of ischemic preconditioning seems to be biphasic, with an early opioid mediated phase occurring within minutes from the initial ischemic insult and lasting for two to three hours, and a late phase becoming apparent 12 to 24 hours later, lasting three to four days and requiring *de novo *protein synthesis[3,4]. The major cellular mechanism responsible for the beneficial effects of ischemic preconditioning seems to be the opening of K_ATP _channels either on the cardiac sarcolemmal or mitochondrial membrane [5-7].

The aim of this study was to explore whether low-dose adenosine infusion exerts an ischemic preconditioning effect in severely symptomatic patients with advanced coronary artery disease.

## Materials and methods

### Patients

We studied nine male patients, mean age 74 years, (range 58-79 years), with severe coronary artery disease (eight with three-vessel and one with two-vessel disease) and severely symptomatic (CCS angina functional class II-III). Except for one patient who had dynpnea, the rest suffered from daily angina. Eight of the patients underwent later coronary bypass grafting (CABG) and one patient with earlier CABG underwent percutanous coronary intervention. All patients had normal global LV systolic function at rest except for one patient who had mildly depressed global LV systolic function (LV ejection fraction ≈ 45%). Three patients had a history of myocardial infarction, three had a history of systemic arterial hypertension, three had a history of paroxysmal atrial fibrillation, three had a mild form of chronic renal failure, and none had a history of diabetes mellitus. All patients were treated with aspirin, beta-blockers, long acting nitrates and calcium channel blockers and seven with either angiotensine converting enzyme inhibitors or angiotensine II receptors blockers.

### Standard echocardiography

We obtained a baseline two-dimensional echocardiography with superimposed Tissue Doppler Echocardiography (TDE) images using a multi-Hertz transducer with commercially available equipment (Vivid 7, GE Vingmed, Horten, Norway) for all patients prior to bicycle exercise stress echocardiography test. Standard parasternal short and long axis views, as well as apical two, three, and four chamber views, were digitized during three consecutive cardiac cycles in cine loop format for off-line analysis.

### Exercise stress echocardiography test protocol

The patients underwent two separate exercise stress echocardiography test in semi-supine position on a specially designed, table-mounted bicycle ergometer. The initial workload was 25 Watts with increments of 10 Watts added every minute. At each stage, the heart rate, systolic blood pressure, and a 12-lead ECG were recorded. We obtained standard echocardiographic images of five consecutive cardiac cycles containing TDE information at rest, sub-maximal workload (heart rate = 70% of the expected maximal heart rate), maximal workload, and recovery phases (one to two minutes after the exercise); all measurements were digitally recorded and stored. Stopping criteria for exercise test was either severe chest pain or achieved 85% of maximal expected heart rate (220-age).

### Study protocol

**Figure 1:**
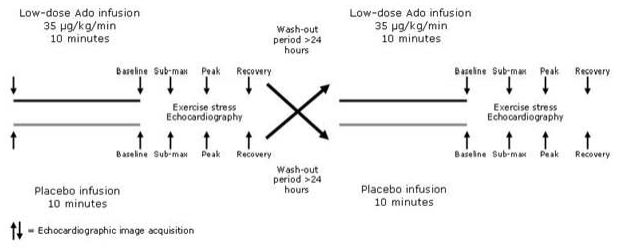
**Study protocol**. The dotted lines represent absence of infusion either of placebo or of adenosine. Echocardiographic images were obtained before adenosine/placebo infusions and at each stage of the exercise test.

An intravenous infusion of either low-dose adenosine (35 μg/kg/min) or placebo (saline infusion) was given in a double-blind fashion over 10 minutes prior to the start of the exercise test. The order of the drug administration was randomly selected and the sequence of infusions was inverted on the second occasion; the first and second occasions occurred at least one day apart, median 3 days (range 1 to 5 days) (Figure 1). The rationale to select this dosage lies on previous studies showing that it had an analgetic effect[8], doses between 50 and 70 μg/kg/min may have a positive effect while doses higher than 70 μg/kg/min provoked ischemia[9] in patients with coronary artery disease.

### Tissue Doppler echocardiography off-line analysis

**Figure 2:**
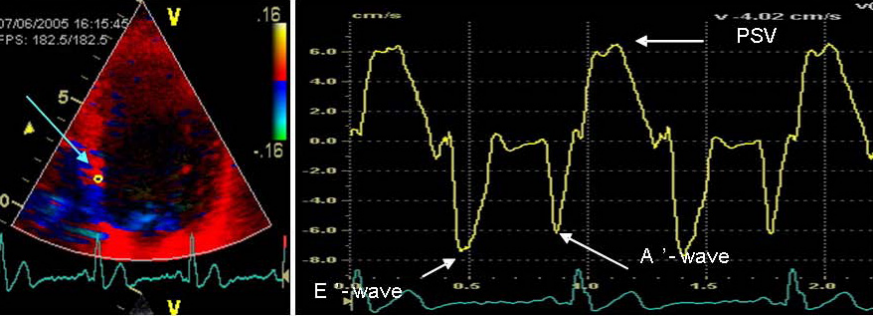
**Tissue Doppler echocardiography**. To the left, an apical four-chamber view picture with the sample volume placed at the basal portion of the septal wall. On the right, all the variables extracted from on-line recording.

The analysis of the echocardiographic images was done by one of the investigators (MQ) blinded to the sequence of drug administration. However, the images corresponding to each stage of the protocol were known as the heart rate at rest and at peak exercise were clearly discernible. A sample volume was positioned on the region of interest at the basal segment of the four LV walls (septal, lateral, inferior, and anterior) to obtain a myocardial velocity profile (Figure 2). We analyzed both the systolic and diastolic phases of the velocity profile. We defined peak systolic velocity (PSV) as the peak velocity during ejection. We also measured peak velocity at early diastole (E'-wave) and peak velocity at the late diastole (A'-wave). The longitudinal A-V plane displacement was calculated by time integration of the PSV. In addition, the degree of myocardial compression/deformation as strain (S) and strain rate (SR) were also calculated.

In order to assess the LV regional systolic function and to detect the presence of regional myocardial ischemia, the LV walls were categorized as ischemic or non-ischemic: a LV wall was defined as ischemic if a reduction, no increment, or an increment of <15% in PSV, was observed during maximal work load compared to baseline preceding placebo-infusion; otherwise, the LV walls were defined as non-ischemic.

### Statistical Analysis

Data are presented as mean ± standard deviation (SD). Statistical comparisons were performed by analysis of variance (ANOVA) with repeated measures. Post hoc comparisons between groups were performed using the Tukey test.

### Ethics

The local Ethics Committee at the Karolinska University Hospital, Huddinge, approved the study. All participants gave written informed consent.

## Results

There were no significant differences in maximal work capacity, hemodynamic parameters including heart rate and blood pressure or ST-depression (Table 1). We observed a significant increment in PSV during maximal stress compared to rest during both the adenosine and placebo infusions; however, we observed no significant differences in PSV between low-dose adenosine and placebo infusions (Table 1).

**Table 1:** Hemodynamic parameters (heart rate, blood pressure), work capacity (Watts) and Tissue Doppler echocardiography variables (expressed as the average of the four LV walls) measured at baseline and maximal work loud during adenosine and placebo infusions

	Adenosine		Placebo	
**Variables**	**Baseline**	**Peak**	**Baseline**	**Peak**

**Heart rate, bpm**	59 ± 11	104 ± 13*	62 ± 14	106 ± 13*
**SBP, mm Hg**	143 ± 12	172 ± 18*	148 ± 20	169 ± 28*
**Watts**	00.0 ± 0.0	83 ± 19	00.0 ± 0.0	83 ± 28
**ST depression**		1.8 ± 1.4		1.4 ± 1.0
**PSV, cm/s**	5.5 ± 1.6	6.4 ± 3.2*	5.5 ± 1.6	7.6 ± 2.6*
**A-V plane, mm**	9.6 ± 2.7	10.9 ± 4.4*	9.0 ± 2.6	11.7 ± 4.3*
**S%**	17.4 ± 5.1	23.4 ± 7.4*	16.5 ± 3.8	22.1 ± 7.2*
**SR**^-1^	-0.9 ± 0.3	-1.9 ± 0.5*	-1.0 ± 0.2	-1.9 ± 1.5*
**E'-wave, cm/s**	6.5 ± 1.9	8.8 ± 4.0*	5.5 ± 2.3	8.5 ± 4.2*
**A'-wave, cm/s**	4.9 ± 2.4	5.1 ± 3.6	4.9 ± 2.5	4.1 ± 2.9

Values are expressed as means ± SD.

Abbreviations: A' = late diastolic A-wave; A-V = atrio-ventricular plane displacement; bpm = beats per minute; E' = early diastolic E-wave; PSV = peak systolic velocity; SBP = systolic blood pressure, S% = strain; SR = strain rate.

* *P *< 0.01 Peak versus baseline

According to the pre-defined criteria, 12 of the 36 LV walls in the nine patients were found to be ischemic during the placebo infusion. When milder criteria were used to define LV wall ischemia, i.e. a reduction, no increment, or an increment of <25% in peak systolic velocity, 15 of the 36 LV walls were found to be ischemic.

**Figure 3:**
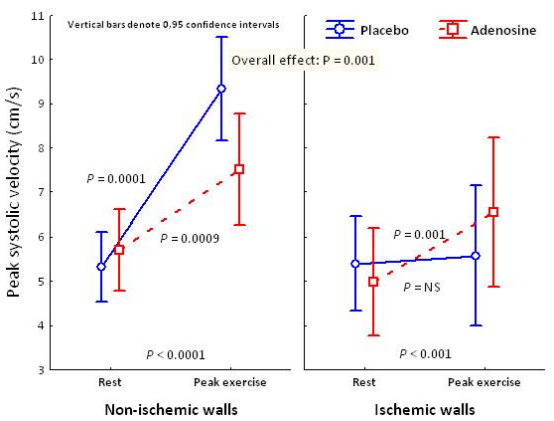
**Peak systolic velocity at rest and peak exercise on ischemic and non-ischemic walls during adenosine and placebo infusions**.

In the ischemic walls, the increment in PSV was higher during adenosine infusion as compared to placebo (*P *< 0.001; Figure 3). The same pattern was seen in AV-plane displacement during systole, and E'-wave (Table 2). There were no significant differences in A'-wave and strain rate according to the ischemic response during low-dose adenosine infusion or placebo (Table 2).

**Table 2:** Tissue Doppler echocardiography of ischemic and non ischemic LV walls during baseline and maximal work loud during low dose adenosine and placebo infusions

	Adenosine				Pacebo			
**Variables**	**Ischemic walls**		**Non ischemic walls**		**Ischemic walls**		**Non ischemic walls**	

	***Rest***	***Peak***	***Rest***	***Peak***	***Rest***	***Peak***	***Rest***	***Peak***

**PSV, cm/s**	5.1 ± 1.4	6.9 ± 2.1*¥	5.6 ± 1.9	7.3 ± 2.7*	6.0 ± 2.0	5.6 ± 1.6	5.4 ± 1.5	9.1 ± 2.7*
**A-V plane, mm**	9.4 ± 2.6	10.3 ± 3.2*¥	10.3 ± 3.7	11.6 ± 5.8*	10.7 ± 2.2	9.7 ± 2.7	8.4 ± 3.2	13.0 ± 5.4*
**S%**	16 ± 3	24 ± 4	16 ± 6	23 ± 12*	17 ± 4	13 ± 5	16 ± 6	27 ± 5*
**SR**^-1^	-1.0 ± 0.9	-1.7 ± 1.3*	-1.4 ± 0.5	-2.0 ± 1.0*	-1.6 ± 0.5	-0.9 ± 1.6	-1.2 ± 0.5	-2.6 ± 1.8*
**E'-wave, cm/s**	7.4 ± 3.7	11.9 ± 5.4*¥	6.4 ± 2.7	10.1 ± 2.4*	7.8 ± 2.3	9.7 ± 5.9*	5.2 ± 2.6	10.4 ± 3.7*
**A'-wave, cm/s**	6.7 ± 1.6	7.3 ± 2.9	4.3 ± 2.6	6.7 ± 1.9*	6.6 ± 1.5	6.1 ± 1.7*	4.0 ± 2.0	7.1 ± 1.5*

Values are expressed as means ± SD.

As in table 1

* *P *< 0.01 Peak versus baseline

¥ *P *< 0.01 Peak - ischemic walls - adenosine, versus peak - ischemic walls - placebo

## Discussion

In this study, a low-dose infusion of adenosine preserved the regional systolic function of LV walls subjected to an exercise-induced ischemic burden. We observed a higher PSV at peak exercise in LV walls rendered ischemic during adenosine infusion than during placebo infusion, suggesting a preconditioning effect of adenosine. The main idea of the study was to investigate the possible preconditioning effect of adenosine infusion in a quantitative manner and thus avoiding any possible bias induced by subjective analysis of wall motion analysis. Therefore, we did not use wall motion score in the diagnosis of myocardial ischemia. The concordance between wall motion core and tissue Doppler imaging in this respect has been reported elsewhere [10]. Having said this, we do not mean that quantitative stress echocardiography is the gold standard for the detection of myocardial ischemia.

Alternative mechanisms for preserving the regional systolic function may have been achieved by adenosine-mediated vasodilatation or by the possible unloading effects of adenosine; however, these are unlikely mechanisms because myocardial ischemia would already have caused maximal vasodilatation and the low-dose adenosine infusion was stopped before the exercise stress test, by which time any previously infused adenosine would have been metabolised and could not have had a vasodilatory effect. In addition, heart rate and blood pressure responses did not differ between the two treatments, excluding the possible unloading effect of adenosine.

Adenosine has been suggested to induce preconditioning[11] by agonizing its A_1 _and A_3 _receptors; however, some of the protective effects of adenosine might occur via an antiplatelet effect or an anti-inflammatory effect that is totally independent of preconditioning. Clinical trials suggest that some preconditioning mimetics reduce myocardial infarct size when given in conjunction with reperfusion [12,13], and may improve survival, especially in selected patients with acute anterior myocardial infarction[14]. It should be noted that the largely neutral effects observed in the AMISTAD study refers to the possible preconditioning effect of adenosine in a different clinical setting, namely the "reperfusion-injury" provoked by opening the infarct related artery in the context of an ST-elevation acute myocardial infarction.

In the present experimental model of exercise induced myocardial ischemia detected by TDE technique in humans, it is impossible to be sure that the reported findings were caused by ischemic preconditioning mediated by the activation of adenosine receptors; however excluding the vascular and unloading effects and considering that infusion of 35 μg/kg/min of adenosine give rise to activation of adenosine receptors[15], the hypothesis of ischemic preconditioning induced by adenosine is highly plausible. Our results are in keeping with Picano et al who by using dipyridamole, which is an adenosine uptake inhibitor reported increased ischemic threshold [16].

Numerous studies have shown that pre-infarction angina pectoris protects patients who develop acute myocardial infarction [17-19], and at least part of this benefit may be due to a preconditioning phenomenon rather than simply recruiting collateral blood flow. Pre-infarction angina pectoris has been associated with smaller myocardial infarct size, decreased prevalence of congestive heart failure, less cardiac death, fewer arrhythmias, and improved cardiac function[18]. Therefore, it might be possible to treat high risk patients or patients with unstable angina pectoris with preconditioning mimetic agents that stimulate the biochemistry of preconditioning without actually causing ischemia.

### Study limitations

The small size of this study precludes the extrapolation of our results to all patients with ischemic heart disease, especially to those with mild forms of exercise-induced ischemia. The positive pre-conditioning effects of adenosine were not observed for all parameters reflecting the regional LV systolic function. In fact, strain rate, a measure of "pure" myocardial contractility, was not affected by adenosine. This result could be explained by the low reproducibility of this measure when assessed during exercise[20].

Certainly, some degree of ischemic preconditioning induced by angina pectoris did exist among all the patients. However, we do not believe that this fact compromised the results of the study, considering the placebo/controlled and the cross-over character of the study. We used high ischemic limit criteria to define myocardial ischemia by tissue Doppler echocardiography, and when milder criteria that allow for the detection of less severe forms of ischemia were applied, the results were the same. However, if we applied the same ischemic criteria as are used in clinical practice[21], almost all LV walls were categorized as ischemic, making it impossible to perform adequate statistical analysis. This fact emphasizes that we were studying patients with severe coronary artery disease: severely symptomatic and with severe exercise induced myocardial ischemia.

### Possible clinical implications

The present results may open the possibilities of testing new therapeutic agents for the treatment of not only high risk patients with preconditioning mimetics that stimulate the biochemistry of preconditioning without actually causing ischemia but also of a vast number of patients with stable angina pectoris with mild to severe symptom in whom coronary revascularisation is impossible or not achievable.

## Conclusion

Low-dose adenosine infusion reduced the ischemic burden and improved the left ventricular regional systolic function in the ischemic walls of patients with exercise-induced myocardial ischemia, confirming the possible preconditioning effect of adenosine in humans.

## Competing interests

The authors declare that they have no competing interests.

## Authors' contributions

BS selected the patients. MQ carried out the stress tests and MQ and BS analysed the data and statistical measurements. MB participated in the stress tests. CS participated in the design of the study and contributed to the manuscript. All authors read and approved the final manuscript.
